# Supplementary figures and images for: Genetic analysis suggests a surface of PAT-4 (ILK) that interacts with UNC-112 (kindlin)
Source: G3 (Bethesda). 2022 May 10;12(7):jkac117. doi: 10.1093/g3journal/jkac117 (PMC9258589; doi:10.1093/g3journal/jkac117)

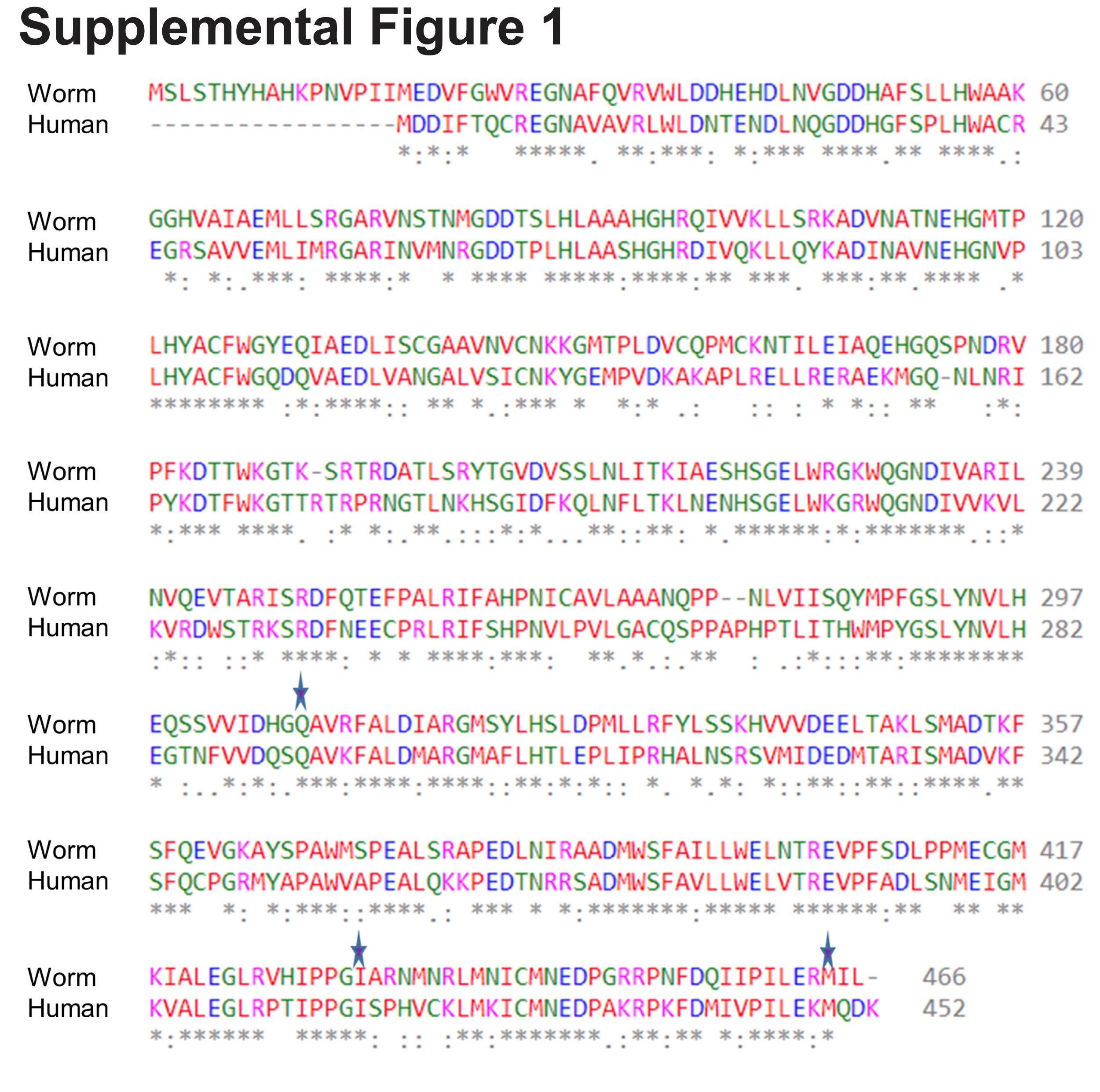

Supplement: jkac117_Supplemental_Figure_1 [file jkac117_supplemental_figure_1.jpeg]

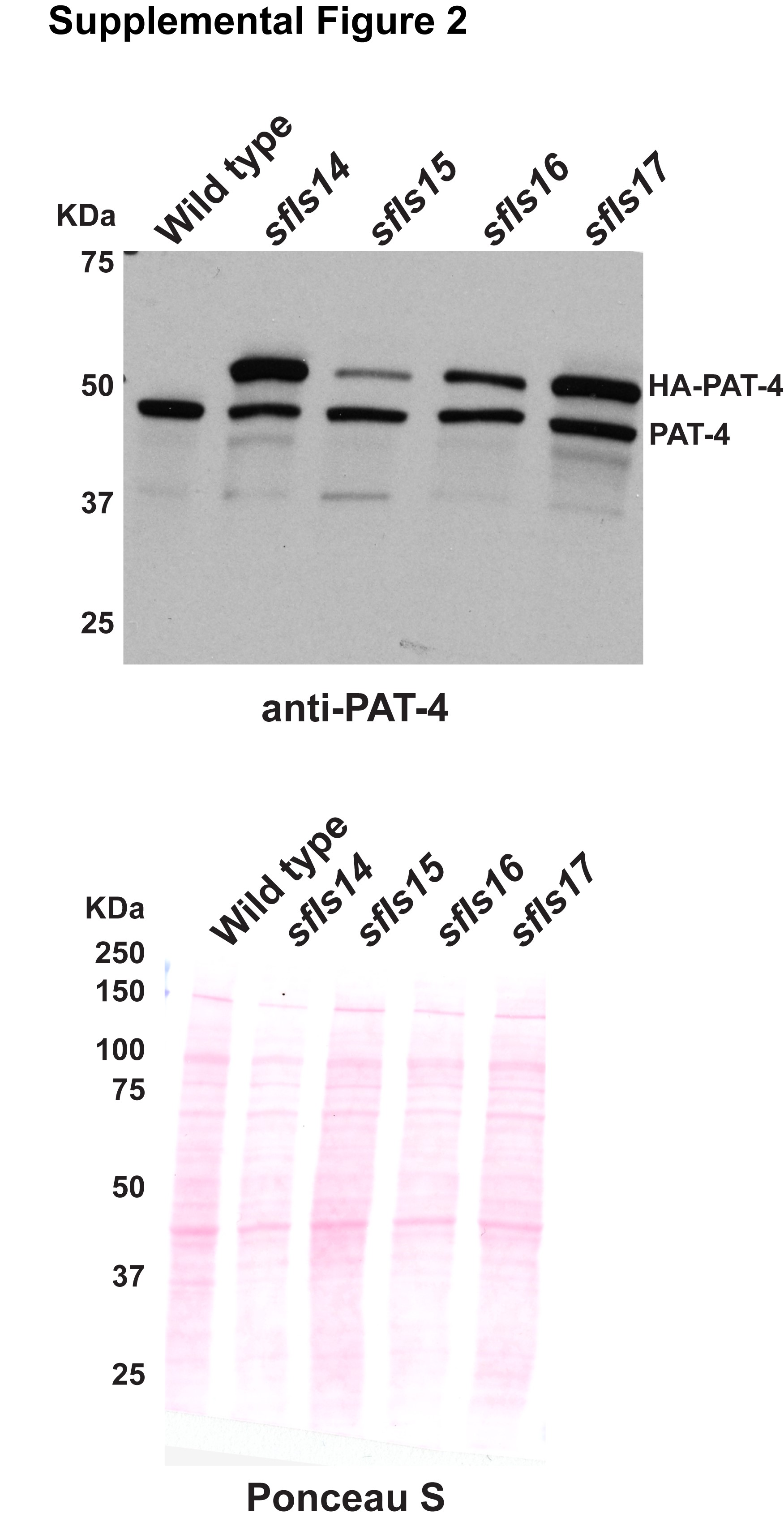

Supplement: jkac117_Supplemental_Figure_2 [file jkac117_supplemental_figure_2.jpeg]
