# Supplementary material for: Genetic analysis suggests a surface of PAT-4 (ILK) that interacts with UNC-112 (kindlin)
Source: G3 (Bethesda). 2022 May 10;12(7):jkac117. doi: 10.1093/g3journal/jkac117 (PMC9258589; doi:10.1093/g3journal/jkac117)
Supplement: jkac117_Supplemental_Material_Legends [file jkac117_supplemental_material_legends.docx]

**Supplemental Figure 1**. Alignment of human ILK and worm PAT-4. The blue stars indicate residues in the PAT-4 pseudo kinase domain that when mutated fail to bind to UNC-112.

**Supplemental Figure 2.** Expression levels of endogenous PAT-4 and HA-PAT-4 in integrated transgenic lines. Approximately equal amounts of total Laemmli-soluble proteins were separated by SDS-PAGE, transferred to a blot (see Ponceau S staining in the bottom panel) and reacted with anti-PAT-4 antibodies and imaged by ECL. See Materials and Methods for description of each line indicated. These transgenic lines express both endogenous PAT-4 (lower band) and heat-shock induced HA-PAT-4 (upper band). In each line, the amount of heat-shock induced HA-PAT-4 is approximately the same as the amount of endogenous PAT-4.
